# Human papillomavirus (HPV) trials: A cross-sectional analysis of clinical trials registries

**DOI:** 10.1080/21645515.2024.2393481

**Published:** 2024-08-28

**Authors:** Edison Johannes Mavundza, Tshiamo Moshading Mmotsa, Duduzile Ndwandwe

**Affiliations:** Cochrane South Africa, South African Medical Research Council, Cape Town, South Africa

**Keywords:** Human papillomavirus, clinical trials, clinical trial registry, cross-sectional analysis, international clinical trials registry platform

## Abstract

Every clinical trial must be registered in a publicly accessible trial registry before enrollment of the first participant. Prospectively registering clinical trials before enrolling participants helps to prevent unethical research misconduct from occurring, duplication of research and increases transparency in research. The aim of this study was to provide cross-sectional survey analysis of planned, ongoing and completed human papillomavirus (HPV) clinical trials conducted worldwide. We searched the International Clinical Trials Registry Platform (ICTR) for registered HPV trials on 5 March 2023. Two authors independently extracted data including name of the clinical trial registry, location of the trial, recruitment status of the trial, gender of participants, phase of the trial, and type of trial sponsor. We used Microsoft Excel to perform descriptive analysis. The search yielded 1632 trials registered between 1999 and 2023. Most of the trials were registered in ClinicalTrials.gov and were registered retrospectively. We also found that most trials were conducted in North America, in recruiting stage, and indicated “not applicable” under the phase of the trial field. Finally, most trials were sponsored by hospitals. Our study found that there are many HPV clinical trials registered in different clinical trial primary registries around the world. However, many of the trials were registered retrospectively instead of the required prospectively and some had missing fields. Therefore, there is a need for registries to promote prospective trial registration and completion of all fields during the registration process.

## Introduction

Human papillomavirus (HPV) infection is the most common sexually transmitted infection in the world.^1,2^ It is estimated that approximately 75% of sexually active men and women acquire HPV infection during their lifetime.^3^ Although most HPV infections are asymptomatic and transient, persistent infection with high-risk HPV types may result in cancers^4^ including anal, cervical, oropharyngeal, penile, vaginal, and vulvar cancer.^3,5–8^ Cervical cancer, the fourth most common cancer in women worldwide, is the most common type of cancer associated with HPV, with an estimated 604,127 cases and 341,831 deaths in 2020.^3^ The low- and middle-income countries (LMICs), especially sub-Saharan Africa carry the severe burden of the disease,^8,9^ accounting for 20% of cases and 25% of deaths from cervical cancer worldwide.^10^ HPV vaccination for women and men, cervical screening (Pap smear) and treatment of precancerous lesions for women are tools used to prevent and control HPV infection and its complications.^5,11–15^

Clinical trials are research studies that are used to evaluate the efficacy and safety of new healthcare interventions in humans.^16–20^ According to Callif 2012,^21^ clinical trials are the central means by which preventive, diagnostic, and therapeutic strategies are evaluated. Clinicians and other decision makers use the results of clinical trials to make informed choices about the benefits and safety of healthcare interventions.^22,23^ There are two main types of clinical trials: interventional and observational.^18,24,25^ Clinical trials have five phases: phase 0, phase 1, phase 2, phase 3, and phase 4.^16,18,25^ Phase 0 trial, which was previously conducted in animals but now is carried out in humans,^25^ is done to determine pharmacokinetics and pharmacodynamics.^18,25^ Phase 1 trials are conducted to evaluate safety and dosage of an intervention, while those in phase 2 are carried out to evaluate safety and efficacy of an intervention. Phase 3 clinical trials are conducted to confirm safety and efficacy of an intervention.^18,20,26^ Phase 4 clinical trials, also called the post-approval or the post-marketing phase studies,^25^ are conducted to monitor the safety and effectiveness of the intervention in the real world conditions.^18,25,26^

Clinical trial registration involves the registration of a clinical trial’s details in a publicly accessible platform before recruitment or enrollment of first participant. The registration of clinical trials provides the public, clinicians, and researchers with the access to the information of all conducted clinical trials.^27,28^ It brings to light the type of research being conducted, where and how it is being conducted, and who is conducting it.^29^ Sharing of information on clinical trials has been recognized as one of the most outstanding achievements in clinical research.^30^ According to Edem 2021,^31^ registration of clinical trial is both an ethical and legal obligation in the conduct of clinical trial. In 2005, the International Committee of Medical Journal Editors (ICMJE) mandated the prospective registration of each clinical trial in a publicly accessible, web-based clinical trial registry.^29,32,33^ The ICMJE also mandated the registration of clinical trial as a prerequisite for the publication of the results.^29,34^ Prospective registration of clinical trials helps to prevent unethical research misconduct from occurring, duplication of research and increase research transparency.^17,29,30,35^ A clinical trial registry is a publicly available online database that contains information about planned, ongoing and completed clinical studies.^30,33,36^ It includes information on study design, conduct, administration, results reporting, and investigator’s data sharing plans.^33^ Clinical trials registers have been in existence for decades, especially in the field of cancer, but have grown in number over the last decade.^36^ Many publicly accessible, web-based clinical trial registries have been established around the world.^37^ In 2005, the World Health Organization (WHO) established the International Clinical Trials Registry Platform (ICTRP) to link these clinical trial registries and provide a single point of access to information on all registered clinical trials around the world.^27,29,34,35,37^ ICTRP bring together data from trials registered in different clinical trial primary registries around the world.^29,34^ The WHO-ICTRP registry network consist of primary and partner clinical trial registries.^27,29^ Primary clinical trial registries are registries which meet specific criteria for content, quality and validity, accessibility, unique identification, technical capacity, and administration.^27,29,33,38^ Although they meet the same of the criteria as primary registries, partner registries are not required to have a regional or national mandate, be managed by a nonprofit organization and accept prospective registration.^27^ Currently, there are 17 primary registries and 1 partner registry (ClinicalTrials.gov) that provide data on registered clinical trials to the ICTRP.^27,29,33,39^ ICTRP is currently recognized as the largest clinical trial platform in the world with more than 700 000 records of clinical trials by 31 October 2021.^33^

To the best of our knowledge, there is no study that evaluated registered HPV clinical trials. The objective of this study was therefore to identify and analyze planned, ongoing, and completed all HPV clinical trials registered in the ICTRP.

## Methods

This study used data obtained from the ICTRP. ICTRP is a one-stop search portal for all registered clinical trials in the primary clinical trial registries of the WHO registry network. Our study is a cross-sectional analysis of the HPV clinical trials registered in the ICTRP. On 5 March 2023, we searched the ICTRP database using the term “human papillomavirus.” The search output was downloaded and exported into Microsoft Excel spreadsheet for deduplication and analysis. We included all HPV clinical trials. Two authors (EJM and TMM) independently screened the titles of the records to select eligible studies. We included all types of HPV clinical trials. We used Microsoft Excel to perform descriptive analysis of name of the clinical trial registry, location of the trial, recruitment status of the trial, gender of participants, phase of the trial, and type of trial sponsor. We considered “not recruiting” clinical trials as those that are done with the recruitment of participants, and “recruiting” clinical trials as those that are actively recruiting participants. We used tables and graphs to present our findings.

## Results

### Results of the search

**Figure 1.**
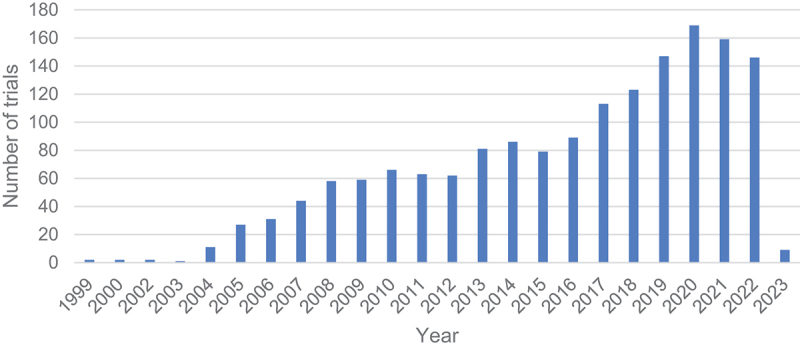
Registered HPV trials by year.

A total of 1632 clinical trials on HPV were identified in the ICTRP database. Of these trials, 1211 (74.2%) were interventional studies, while 421 (25.8%) were observational studies. The trials were registered between 1999 and 2023, with many of them (*n* = 169) registered in 2020 (Figure 1). Most of the clinical trials (65.8%, *n* = 1075) were registered retrospectively, while the remaining 558 (34.2%) were registered prospectively. The number of clinical trials registered per registry are shown in Table 1. We found that most clinical trials (68.0%, *n* = 1109) were registered in ClinicalTrials.gov, while the least (0.3%, *n* = 5) were registered in the Pan African Clinical Trial Registry (PACTR).

**Table 1.** Number of clinical trials per registry.

Registry	N	%
ANZCTR	37	2.3
ChiCTR	81	5.0
ClinicalTrials.gov	1109	68.0
CRIS	15	0.9
CTRI	53	3.2
GermanCTR	15	0.9
EU-CTR	104	6.4
IRCT	14	0.9
ISRCTN	75	4.6
ITMCTR	7	0.4
JPRN	57	3.5
NTR	26	1.6
PACTR	5	0.3
ReBec	6	0.4
REPEC	18	1.1
TCTR	10	0.6
**Total**	**1632**	**100.0**

ANZCTR, Australian New Zealand Clinical Trials Registry; ReBec, Brazilian Clinical Trials Registry; ChiCTR, Chinese Clinical Trial Registry; Clinical CRIS, Research Information Service; CTRI, Clinical Trials Registry - India; GermanCTR, German Clinical Trials Register; EU-CTR, EU Clinical Trials Register; IRCT, Iranian Registry of Clinical Trials; ISRCTN, International Standard Randomised Controlled Trial Number; ITMCTR, International Traditional Medicine Clinical Trial Registry; JPRN, Japan Primary Registries Network; NTR, Netherlands Trial Register, PACTR, Pan African Clinical Trial Registry; REPEC, Peruvian Clinical Trial Registry; TCTR, Thai Clinical Trials Registry.

### Location of the trials

**Figure 2.**
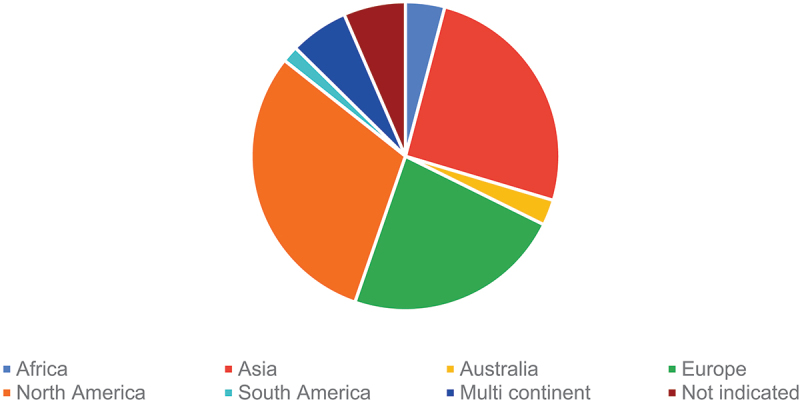
Geographic distribution of HPV clinical trials.

Figure 2 shows the geographical distribution of HPV clinical trials around the world. Our findings revealed that most trials were conducted in North America (30.4%, *n* = 495), followed by Asia (25.5%, *n* = 416), Europe (23.0%, *n* = 375), Africa (4.1%, *n* = 67), Australia (2.7%, *n* = 44), and South America (1.7%, *n* = 28). Hundred (6.1%) trials were conducted in multi continent, while the remaining 107 (6.6%) did not indicate the location of the trial.

### Recruitment status of the trials

The recruitment status of trials is shown in Table 2. We found that most HPV clinical trials (71.2%, *n* = 1162) were not recruiting, followed by 400 (25.7%) which were recruiting. We also found that 6 (0.4%) clinical trials did not indicate their recruitment status. Majority of the trials were conducted among females (55.2%, *n* = 900), followed by both females and males (35.1%, *n* = 572), males (4.7%, *n* = 76), while the remaining 84 (5.2%) did not indicate the gender of the participants.

**Table 2.** Recruitment status of the HPV clinical trials.

Recruitment status	N	%
Authorised	38	2.3
Not recruiting	1162	71.2
Recruiting	420	25.7
Not applicable	6	0.4
Not indicated	6	0.4
**Total**	**1632**	**100.0**

### Phase of the trials

Our study found that majority of clinical trials (49.8%, *n* = 813) had “not applicable” under phase of trial, while 86 (5.3%) did not indicate their phase. Majority of those indicated their phases were in phase 3 (12.8%, *n* = 209), followed by phase 2 (12.3%, *n* = 200), and phase 1 (7.1%, *n* = 115). We also found that the were 91 (5.6%) registered HPV clinical trials in phase 4 (Table 3).

**Table 3.** Phase of registered HPV clinical trials.

Phase	N	%
Phase 0	43	2.6
Phase 1	115	7.1
Phase 1/phase 2	60	3.7
Phase 2	200	12.3
Phase 2/phase 3	15	0.9
Phase 3	209	12.8
Phase 4	91	5.6
Not applicable	813	49.8
Not indicated	86	5.3
**Total**	**1632**	**100.0**

### Sponsor of the trials

Table 4 is showing the sponsors of the HPV clinical trials. We found that most trials (29.8%, *n* = 486) were sponsored by hospitals, followed by universities (27.8%, *n* = 453), and pharmaceutical companies (21.7%, *n* = 353). We also found that 88 (5.4%) trials were self-funded, while 4 (0.3%) did not receive any funding.

**Table 4.** Sponsors of registered HPV clinical trials.

Type of sponsor	N	%
Governmental	88	5.4
University	453	27.8
Pharmaceutical	353	21.7
Self-funding	88	5.4
No funding	4	0.3
Funding agency	11	0.7
Research institute	107	6.6
Non-profit organisation	37	2.3
Hospital	486	29.8
Multi funders	2	0.1
Not indicated	3	0.1
**Total**	**1632**	**100.0**

## Discussion

In this paper, we conducted a descriptive analysis of the HPV clinical trials in the ICTRP. We found that there were 1632 HPV clinical trials registered between 2000 and 2020. Our findings revealed that most of the trials were registered in the ClinicalTrials.gov registry. Established in 2000 by the US National Institute of Health (NIH) Library of Medicine,^33,39^ ClinicalTrials.gov is the largest clinical trial registry, with 391 704 records on 11 October 2021.^33^ Although clinical trials should be prospectively registered,^29,34,40^ we found that most of the HPV clinical trials were registered retrospectively. Despite being mandatory to register each clinical trial prospectively, some sponsors register their clinical trials after they have started.^27,29^ The registration of clinical trials prospectively helps to prevent selective reporting of trial outcome.^17,29,35^ According to Viergever 2011,^35^ some sponsors of clinical trials conceal negative findings when they are reporting the results of the clinical trials. In addition to the registration of a clinical trial being set as a prerequisite to publish trial’s findings,^27,34^ sponsors are also mandated to publish findings in a peer-reviewed journals to improve transparency.^27^ Therefore, the need to publish trial findings might be the reason most of the HPV clinical trials were registered retrospectively.

Our study found that most of the HPV clinical trials (30.4%, *n* = 495) were conducted in North America, while Africa, which carries the severe burden of the diseases, had only 67 clinical trials conducted. Although there is an increase in the number of clinical trials conducted in Africa, they remain less when compared to the rest of the world.^27^ Since Africa consists mostly of LMICs, low number of clinical trials being conducted may be attributed to the lack of resources in the continent, especially funding to finance clinical trials. Africa is considered the poorest continent in the world.^41^ Most of the countries in Africa are facing difficult economic situations and are characterized by extreme poverty.^42^ Therefore, funding of research in the African continent is a challenge.^43^ In addition, of these 67 trials conducted in Africa, there were only 5 trials that were registered in PACTR, the only WHO primary registry in Africa.

The recruitment status of a trial indicates the stage of the trial.^44^ It shows whether a trial is planned, ongoing or completed. The recruitment status also determines the availability of results for evidence-based decisions.^30^ In this study, we found that 71.2% of the HPV clinical trials were not recruiting, meaning that these were planned trials, while 25.7% trials had recruitment status “recruiting” indicating that these trials were ongoing. Our findings also revealed that most trials were conducted among females. These findings are not surprising at all since sexually active young women are known to carry the highest risk of HPV infection.^45^ Despite this, cervical cancer, one of the most common cancers in women, is the most common cancer associated with HPV infection.^5^ With regards to the phase of the registered clinical trials, we found that most of the trials (49.8%) indicated “not applicable.” There may be two reasons behind this finding. Firstly, of the 1632 HPV clinical trials registered, we found that 25.8% of them were observational studies. Secondly, we found that 71.2% were not yet started, they were planned trials. When exploring the sponsor type of the trials, we found that most of the trials (29.8%) were funded by hospitals. The sponsor of a clinical trial has a responsibility to register a trial.^44^ The sponsor of a clinical trial is an individual, company, institution, or organization which takes responsibility for the initiation, management, and/or financing of a clinical trial. ^46–48^

Some of our findings in this study have both commonalities and dissimilarities with the findings of other similar studies. Similar to our findings, two studies that analyzed registered clinical trials of Cholera^30^ and Rotavirus vaccines^44^ also found that most of the trials were registered with ClinicalTrials.gov primary registry and they were not recruiting. Our finding that most clinical trials were registered retrospectively was also found by Ndwandwe and colleagues.^44^ Different to our finding that most clinical trials were conducted in North America, both studies by Ndwandwe and colleagues^44^ and Mathebula and colleagues^30^ found that most clinical trials were conducted in Asia. Both studies also found that most of the registered clinical trials were in phase 3^44^ and phase 2,^30^ while we found that most of the HPV clinical trials indicated “not applicable” on the phase of the trial. Unlike our study, which found that most of clinical trials were sponsored by hospitals, similar studies reported that most of the Cholera vaccine clinical trials^30^ and Rotavirus vaccine clinical trials^44^ were sponsored by research organizations and pharmaceutical companies, respectively.

Our study has some limitations. Therefore, our findings should be interpreted with caution. The first limitation of our study is that we searched for HPV clinical trials conducted worldwide only on the WHO ICTRP. This means that there may be other HPV clinical trials registered in other registries that are not providing data to the ICTRP that we may have missed. Second limitation of our study is that many registered clinical trials had missing fields. This may have had an influence in our findings in this study. The problem of incomplete fields in registered clinical trials remains a challenge across different registries.^27^

## Conclusion

Our study found that there are many HPV clinical trials registered in different clinical trial primary registries around the world. However, many of the trials were registered respectively instead of the required prospectively and some had missing fields. To increase research transparency, prevent duplication of research, and prevent publication bias, there is therefore an urgent need for all primary registries to make prospective registration mandatory and remains the only type of registration available to clinical trial sponsors. In addition, all clinical trial primary registries should also make completion of all fields compulsory when registering a trial, to increase transparency. Of all registered HPV clinical trials, only a few were conducted in LMICs, countries carrying the highest burden of HPV and its associated diseases. Therefore, there is a need for sponsored to fund more HPV clinical trials in LMICs.
